# Blood pressure at age 40 and key features of cerebral small vessel disease at age 70: data from the ACE 1950 Study

**DOI:** 10.1186/s12872-025-05140-6

**Published:** 2025-10-09

**Authors:** Marte M. Walle-Hansen, Guri Hagberg, Marius Myrstad, Trygve Berge, Thea Vigen, Hege Ihle-Hansen, Bente Thommessen, Inger Ariansen, Magnus N. Lyngbakken, Helge Røsjø, Ole M. Rønning, Mona K. Beyer, Arnljot Tveit, Håkon Ihle-Hansen

**Affiliations:** 1 Department of Medical Research, Bærum Hospital, Vestre Viken Hospital Trust, Sogneprest Munthe-Kaas Vei 100, Gjettum, 1346 Norway; 2 Institute of Clinical Medicine, University of Oslo, Oslo, Norway; 3 Stroke Unit, Department of Neurology, Oslo University Hospital, Ullevål, Norway; 4 Department of Internal Medicine, Bærum Hospital, Vestre Viken Hospital Trust, Drammen, Norway; 5 Department of Neurology, Division of Medicine, Akershus University Hospital, Lørenskog, Norway; 6 Department of Chronic Diseases, Norwegian Institute of Public Health, Oslo, Norway; 7 Department of Cardiology, Division of Medicine, Akershus University Hospital, Lørenskog, Norway; 8 K.G. Jebsen Center for Cardiac Biomarkers, Institute of Clinical Medicine, University of Oslo, Oslo, Norway; 9 Akershus Clinical Research Center (ACR), Division of Research and Innovation, Akershus University Hospital, Lørenskog, Norway; 10 Division of Radiology and Nuclear Medicine, Oslo University Hospital, Oslo, Norway; 11 Department of Acute Medicine, Oslo University Hospital, Oslo, Norway

**Keywords:** Hypertension, Elevated blood pressure, Cerebral small vessel disease, Magnetic resonance imaging, Risk factors, Longitudinal studies

## Abstract

**Background:**

The separate effects of systolic (SBP) and diastolic blood pressure (DBP) on cerebral small vessel disease (cSVD) development needs elucidation. We investigated the association between SBP and DBP at age 40 and two selected brain magnetic resonance imaging (MRI) features of cSVD (lacunes and white matter hyperintensities [WMHs]) at age 70 in a general Norwegian population cohort.

**Methods:**

We included individuals from the Akershus Cardiac Examination (ACE) 1950 Study (2012–2015) who had previously participated in the Age 40 Program (1990–1993).

A random subset of participants with SBP in the categories of non-elevated (< 120 mmHg), high elevated (130–139 mmHg) or hypertension (≥ 140 mmHg) at age 40 were invited to perform brain MRI for assessment of cSVD (lacunes and WMHs) at age 70 (2016–2024). DBP was categorized as non-elevated (< 70 mmHg), low elevated (70–79 mmHg), high elevated (80–89 mmHg) and hypertension (≥ 90 mmHg). Logistic and ordinal regressions assessed the association between SBP and DBP and lacunes and severity of WMHs (measured with Fazekas scale), adjusting for sex, total cholesterol, smoking, physical activity, diabetes, education, and age at MRI, with non-elevated BP as the reference category.

**Results:**

A total of 414 participants (167 [40%] women) were included. Participants were 70.2 ± 2.3 years when undergoing brain MRI. Mean Fazekas scale was 1.3 ± 0.8, and 54 (13%) had lacunes. SBP and DPB were not associated with lacunes. DPB of 80–89 mmHg (adjusted OR [95% CI], 1.91 [1.01–3.62]) and ≥ 90 mmHg (2.11 [1.06–4.19]) at age 40 were associated with WMHs.

**Conclusions:**

Elevated DBP (80–89 mmHg) and diastolic hypertension (> 90 mmHg) at age 40 were associated with WMH burden at age 70, suggesting a long-term association between midlife DBP and cerebrovascular health.

**Trial registration:**

ClinicalTrials.gov. NCT01555411. 15 March 2012.

**Graphical Abstract:**

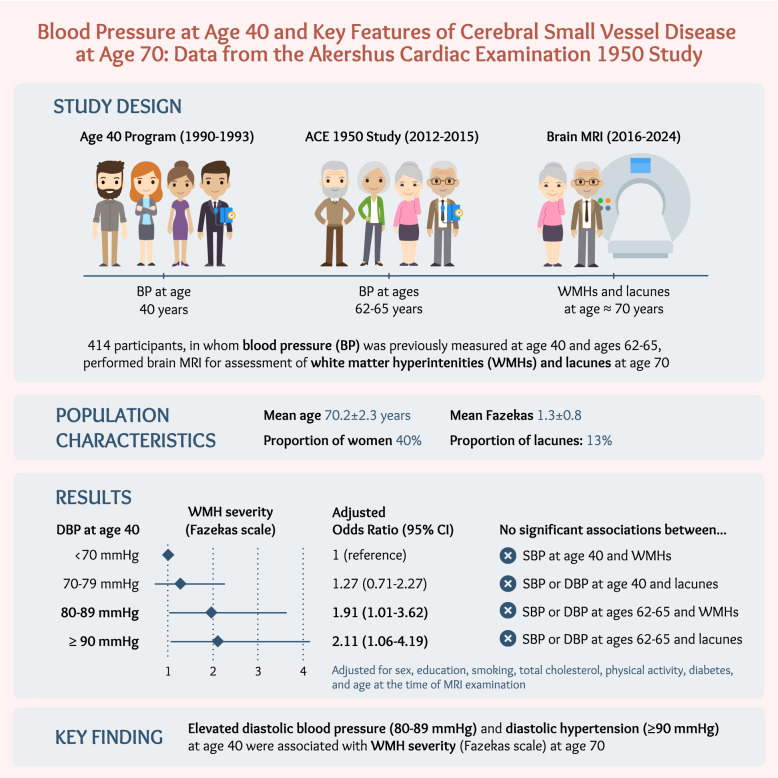

Figure developed by the authors using venngage.com with a license to use, reproduce and distribute worldwide. ACE, Akershus Cardiac Examination. BP, blood pressure. DBP, diastolic blood pressure. MRI, magnetic resonance imaging. WMHs, white matter hyperintensities.

**Supplementary Information:**

The online version contains supplementary material available at 10.1186/s12872-025-05140-6.

## Background

Cerebral small vessel disease (cSVD) is a common ageing phenomenon [1–3], and is associated with an increased risk of cerebrovascular events, cognitive decline [4–7], and dementia [8–11]. Increased blood pressure is associated with cSVD development, with neuroimaging features including lacunes and white matter hyperintensities (WMHs) [12], and current literature acknowledges hypertension as a key risk factor for cSVD [13, 14]. Previous research indicates that elevated diastolic blood pressure in early midlife may be particularly damaging to the cerebral microcirculation [15], but previous studies have predominately used systolic blood pressure [16] or combined systolic and diastolic blood pressure as the main exposures [1, 17, 18]. Thus, investigations on the separate associations between systolic and diastolic blood pressure in early midlife and subsequent cSVD development are warranted.

The latest European Society of Cardiology guidelines on the management of elevated blood pressure and hypertension (2024) recommend blood pressure screening from the age of 40 years [19]. However, the clinical implications of these guidelines, including the role of early blood pressure screening to identify individuals at risk of subclinical and clinical future cSVD, have not been fully elucidated.

Consequently, in the present study, we aimed to describe the separate associations between systolic and diastolic blood pressure at age 40 and two neuroimaging features of cSVD (lacunes and WMHs) at age 70 in a cohort from the general Norwegian population. Secondly, we examined how blood pressure trajectories, both systolic and diastolic, between age 40 and the mid-60s influenced the presence and severity of these features at age 70.

## Methods

### Design and setting

**Fig. 1:**
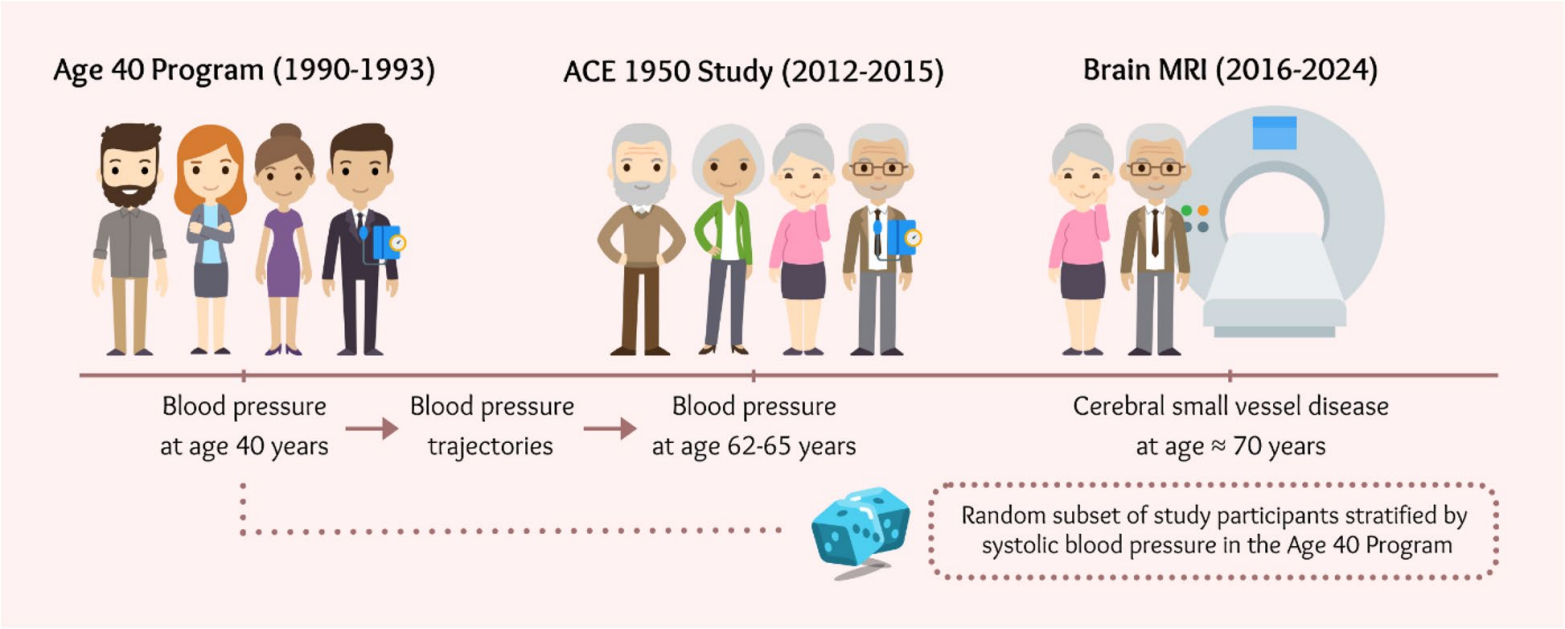
Study design. Legend: Figure developed by the authors using venngage.com with a license to use, reproduce and distribute worldwide. ACE, Akershus Cardiac Examination. MRI, magnetic resonance imaging

In this study, we included individuals from the ACE 1950 Study who had previously participated in the Age 40 Program. The main exposure, blood pressure at age 40, was measured during the Age 40 Program. Blood pressure trajectories from age 40 to the mid-60s were defined as changes in blood pressure between the Age 40 Program (1990–1993) and the ACE 1950 Study first visit (2012–2015). The two main outcomes, the presence of lacunes and the severity of WMHs according to Fazekas scale, were assessed by brain magnetic resonance imaging (MRI) performed on a random subset of the study participants at age 70. This study adheres to the STROBE (Strengthening The Reporting of OBservational studies in Epidemiology) guidelines. Figure 1 demonstrates the study design.

### The Akershus Cardiac Examination 1950 Study (2012–2015)

The ACE 1950 Study is a prospective cohort study of men and women born in 1950 living in Akershus County in Norway. During the first visit conducted between 2012 and 2015, a total of 3706 individuals underwent an extensive cardiovascular examination including a fasting blood sample, a clinical examination, and a self-administered questionnaire on medical history and health behaviour. Details on the measurement procedures during the first visit have been previously published [20]. Briefly, after 5 min of rest, blood pressure was measured three times in the seated position, with the arm supported at heart level using an automated device (Carescape V100, GE Healthcare). At least 10 s intervals were kept between each blood pressure reading, and the mean of the second and third reading was used in statistical analysis. The ACE 1950 Study protocol and characteristics of the cohort have been previously published [20, 21].

### The Age 40 Program (1990–1993)

Between 1985 and 1999, Norwegian Health Authorities conducted The Age 40 Program, a nationwide screening examination of the cardiovascular health of 40-year old men and women living in Norway [22]. All individuals aged 40 years were invited to participate. In Akershus County (1990–91), among 40-year-olds (born 1948–1950), attendance rate was 69%. Among the 3706 participants in the ACE 1950 Study, 2733 (76%) also participated in the Age 40 Program between 1990 and 1993. The Age 40 program included a non-fasting blood sample, a clinical examination, and a self-administered questionnaire on medical history and health behaviour. Details on the collection of clinical variables in the Age 40 Program have been described previously [22]. We used the mean of the second and third blood pressure readings, obtained after three consecutive measurements in the seated position following 2 min of rest, with 1-min intervals between each reading, using an automated device (DINAMAP, Criticon, Tampa, Florida, USA).

### Study population

Participants were selected into the present study as follows: The starting population was defined as all individuals who had participated in the ACE 1950 Study and the Age 40 Program, with available blood pressure measurements in both studies. These individuals were categorized into four groups according to systolic blood pressure in the Age 40 Program ([1] less than 120 mmHg, [2] 120–129 mmHg, [3] 130–139 mmHg and [4] at least 140 mmHg). Of the following three SBP categories – [1] less than 120 mmHg, [3] 130–139 mmHg and [4] at least 140 mmHg, participants were randomly selected and invited to undergo brain MRI. The selection process involved multiple rounds of invitation by mail. In each round, a total of 20 individuals from each of the three SBP categories were randomly selected (60 in total), and an invitation letter was sent by standard mail. Individuals who provided written informed consent were referred for MRI and included in the study. This invitation cycle was repeated until the target number of participants had been reached. Individuals with systolic blood pressure in the category of 120–129 mmHg were excluded in order to invite a larger number of individuals from the groups of particular interest.

### Brain MRI protocol (2016–2024)

Brain MRIs were performed between 18 November 2016 and 10 February 2024. A predefined MRI protocol for visual evaluations of lacunes and WMHs was used. Visual assessment of WMHs has shown good inter- and intrarater agreement [23]. Image acquisition was made using a 3-Tesla MR machine (Siemens) with the following sequence protocol: sagittal T2-weighted Fluid-Attenuated Inversion Recovery (FLAIR), antero-posterior EP2D Diffusion 64 DTI Isotropic slices of 2-mm thickness, Restrictive Sensitivity Imaging, T2-weighed TSE Transverse slices of 3-mm thickness, and sagittal T1-weighed Magnetization-Prepared Rapid Acquisition Gradient Echo (MPRAGE). The majority of scans (94%) were interpreted by an experienced neuroradiologist, while the remaining ones were interpreted by an experienced radiologist.

### Outcomes

The present study had two main outcomes as markers of cSVD: the presence of lacunes and the severity of WMHs on brain MRI. Lacunes were defined according to current guidelines [12], as fluid-filled round cavities < 15 mm in size, located in white matter, visible on FLAIR MRI sequencing. The severity of WMHs was graded using the modified Fazekas scale, a visual rating scale widely used in the clinical setting, based on FLAIR imaging sequences, with a score ranging from 0 (no WMHs) to 3 (severe degree of WMHs) [24]. Among healthy individuals, the presence of a lacune is associated with more than a twofold increase in the future risk of stroke [25].

### Exposures

The main exposures, systolic and diastolic blood pressure at age 40 years, were obtained in the Age 40 Program (1990–1993) and categorized as the following: < 120 mmHg (non-elevated), 130–139 mmHg (high elevated), and ≥ 140 mmHg (hypertension) for systolic blood pressure, and < 70 mmHg (non-elevated), 70–79 mmHg (low elevated), 80–89 mmHg (high elevated) and ≥ 90 mmHg (hypertension) for diastolic blood pressure [19]. Blood pressure in the mid-60s was obtained in the ACE 1950 Study first visit (2012–2015), when participants were 62–65 years old. Systolic and diastolic blood pressure categories at age 40 were classified solely on the basis of blood pressure measurements, independent of self-reported history of hypertension or antihypertensive medication use.

The secondary exposures, systolic and diastolic blood pressure trajectories between age 40 and the mid-60s were defined into six groups, based on blood pressure measurements and self-reported hypertension, as well as self-reported use of antihypertensive medication: (1) *Non-elevated systolic blood pressure throughout* (a systolic blood pressure of no more than 120 mmHg in the Age 40 Program and in the ACE 1950 Study first visit), (2) *High elevated systolic blood pressure to systolic hypertension* (a systolic blood pressure of 130–139 mmHg in the Age 40 Program and at least 140 mmHg or use of antihypertensive medication in the ACE 1950 Study first visit), (3) *Systolic hypertension throughout* (a systolic blood pressure of at least 140 mmHg or self-reported hypertension in the Age 40 Program and a systolic blood pressure of at least 140 mmHg or the use of antihypertensive medication in the ACE 1950 Study first visit), (4) *Non-elevated diastolic blood pressure throughout* (a diastolic blood pressure of no more than 70 mmHg in the Age 40 Program and in the ACE 1950 Study first visit), (5) *Elevated diastolic blood pressure to diastolic hypertension* (a diastolic blood pressure of 70–89 mmHg in the Age 40 Program and at least 90 mmHg or the use of antihypertensive medication in the ACE 1950 Study first visit), and (6) *Diastolic hypertension throughout*, (a diastolic blood pressure of at least 90 mmHg or self-reported hypertension in the Age 40 Program and a diastolic blood pressure of at least 90 mmHg or the use of antihypertensive medication in the ACE 1950 Study first visit).

Use of antihypertensive medication was defined as the self-reported use of medications from at least one of the following Anatomical Therapeutic Chemical Classification codes: C02 (antihypertensives), C03 (diuretics), C07 (beta blocking agents), C08 (calcium channel blockers) or C09 (agents acting on the renin-angiotensin system).

### Other covariates

Other covariates from the Age 40 Program and the ACE 1950 Study first visit were defined according to their respective study protocols [20, 22]. Total cholesterol was defined as mmol per liter (mmol/L). Daily smoking and diabetes were self-reported in a questionnaire. Physical activity levels were self-reported using the Saltin-Grimby Physical Activity Level questionnaire [26]*.* Body mass index (BMI) was defined as weight in kilograms divided by the square of height in meters. Obesity was defined as a BMI of at least 30 kg/m^2^. Education was self-reported via a questionnaire as the number of years of formal education at the first ACE 1950 Study visit. A detailed description of covariate measurements is shown in Supplemental Table S1.

### Statistical analysis

We summarized baseline characteristics using means with standard deviations (SDs) for continuous variables and numbers with percentages for categorical variables across categories of systolic blood pressure at age 40. Comparisons of continuous data were made using the Student’s t test or one-way ANOVA. Categorical data were compared using the χ2 test.

We used logistic regression to calculate odds ratios (ORs) with 95% confidence intervals (CI) for the presence of lacunes, and ordinal regression to calculate ORs with 95% CI for the severity of WMHs, assessed by the Fazekas scale, according to categories of systolic and diastolic blood pressure at age 40. Systolic and diastolic blood pressure trajectories between age 40 and the mid-60s were also investigated as categorical exposures to the main outcomes. In each analysis, the lowest blood pressure category was used as the reference group. Results from the regressions are presented as crude models (adjusted for the participant’s age at the time of the MRI examination) and fully adjusted models. The fully adjusted models include a predefined set of covariates from the Age 40 Program: sex, total cholesterol, and the self-reported measures of daily smoking, physical activity, and diabetes, as well as self-reported education obtained in the ACE 1950 Study first visit, and age at MRI. We performed a sensitivity analysis in which hypertension was defined solely by blood pressure readings, regardless of antihypertensive medication use and self-reported hypertension. In a separate sensitivity analysis, we excluded physical activity as a covariate due to its debated impact on cSVD [27, 28].

Two-sided P values below 0.05 were considered statistically significant. Analyses were performed using Stata version 17 (StataCorp).

## Results

### Participant characteristics

**Fig. 2:**
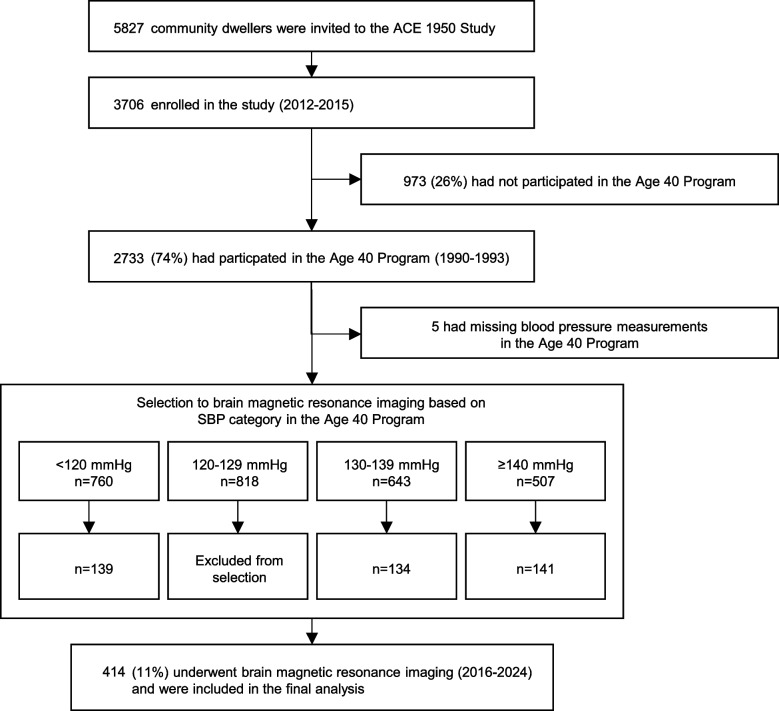
Inclusion of participants to the study. Footnote: ACE 1950; Akershus Cardiac Examination 1950 Study, SBP; systolic blood pressure

A total of 5827 individuals living in Akershus County by 1 November 2011 were considered eligible to participate in the ACE 1950 Study. Of these, 3706 enrolled in the study (attendance rate 64%). Between November 2016 and February 2024, 414 of the 3706 (11%) participants underwent brain MRI. None of these individuals had a history of stroke before age 40, and all were included in the present study (Fig. 2).

Their mean age ± SD was 40.1 ± 0.3 and 167 (40%) were women. The prevalence of cardiovascular comorbidity was low, with 7 (2%) individuals reporting a history of hypertension. A total of 134 (32%) participants had high elevated systolic blood pressure (130–139 mmHg), and 141 (34%) had systolic hypertension (≥ 140 mmHg), while 122 (29%) had high elevated diastolic blood pressure (80–89 mmHg), and 73 (18%) had diastolic hypertension (≥ 90 mmHg).

Participants were 70.2 ± 2.3 years at the time of brain MRI. A total of 54 (13%) participants had lacunes and the mean Fazekas scale was 1.3 ± 0.8. Study population characteristics at age 40 and in the mid-60s are demonstrated in Table 1, and detailed characteristics and sex-specific population characteristics are provided in Supplemental Table S2. The participants who underwent brain MRI were considered representative of the overall ACE 1950 Study population. Supplemental Table S3 provides characteristics of individuals from the ACE 1950 Study with and without brain MRI.

**Table 1:** Population characteristics by systolic blood pressure at age 40, *n* = 414

	**Systolic blood pressure at age 40 years**				
	**Non-elevated** ** < 120 mmHg**	**High elevated** ** ≥ 130 mmHg**	**Hypertensive** ** ≥ 140 mmHg**	**Total**	***P* value**
N (%)	139 (34)	134 (32)	141 (34)	414 (100)	-
Age 40 Program					
Age, years, mean ± SD	40.1 ± 0.4	40.1 ± 0.3	40.1 ± 0.3	40.1 ± 0.3	0.425
Female	102 (73)	37 (28)	28 (20)	167 (40)	< 0.01
Body mass index^a^, kg/m^2^, mean ± SD	23.5 ± 3.2	25.1 ± 2.8	25.2 ± 2.8	24.6 ± 3.0	< 0.01
Blood pressure, mm Hg, mean ± SD					
Systolic	112.4 ± 6.2	134.5 ± 2.7	146.9 ± 7.0	131.3 ± 15.5	< 0.01
Diastolic	70.7 ± 5.3	81.3 ± 7.9	87.5 ± 8.4	79.8 ± 10.1	< 0.01
Sedentary physical activity	28 (20)	22 (16)	20 (14)	70 (17)	0.126
Current or previous smoking^b^	81 (61)	76 (59)	78 (57)	235 (59)	0.760
Total cholesterol, mmol/L, mean ± SD	5.2 ± 0.9	5.7 ± 1.1	5.7 ± 1.1	5.5 ± 1.1	< 0.01
Cardiovascular comorbidity					
Myocardial infarction or angina	1 (1)	0 (0)	1 (1)	2 (0.5)	0.618
Hypertension^c^	0 (0)	0 (0)	7 (5)	7 (2)	< 0.01
Diabetes	0 (0)	0 (0)	0 (0)	0 (0)	-
ACE 1950 Study					
Age, years, mean ± SD	63.9 ± 0.7	63.9 ± 0.6	63.9 ± 0.6	63.9 ± 0.7	0.931
Body mass index, kg/m^2^, mean ± SD	26.7 ± 4.3	27.4 ± 4.0	27.3 ± 3.3	27.1 ± 3.9	0.321
Higher education^d^	71 (51)	60 (45)	67 (48)	198 (48)	0.581
Blood pressure, mm Hg, mean ± SD					
Systolic	130.4 ± 15.7	139.3 ± 15.6	145.5 ± 17.1	138.4 ± 17.3	< 0.01
Diastolic	73.3 ± 8.9	79.3 ± 8.8	82.1 ± 9.6	78.3 ± 9.8	< 0.01
Physically active at least 30 min/day^e^	117 (85)	117 (89)	118 (84)	352 (86)	0.393
Current or previous smoking^f^	86 (62)	86 (64)	85 (60)	257 (62)	0.696
Diagnosed with hypertension	47 (34)	92 (69)	117 (83)	256 (62)	< 0.01
Medication use					
Cholesterol lowering medication	31 (22)	38 (28)	44 (31)	113 (27)	0.234
Antihypertensive medication	21 (15)	56 (42)	76 (54)	153 (37)	< 0.01
Diabetes	7 (5)	11 (8)	18 (13)	36 (9)	0.070
Neuroimaging features of cSVD					
Age at MRI, years, mean ± SD	70.1 ± 2.2	70.2 ± 2.4	70.3 ± 2.2	70.2 ± 2.3	0.704
Fazekas scale^g^					0.785
0: None or a single punctate lesion	14 (10)	14 (10)	13 (9)	41 (10)	
1: Multiple punctate lesions	87 (63)	76 (57)	80 (57)	243 (59)	
2: Beginning confluence (bridging)	24 (17)	27 (20)	25 (18)	76 (18)	
3: Large confluent areas	13 (9)	17 (13)	22 (16)	52 (13)	
Fazekas scale ≥ 2	37 (27)	44 (33)	47 (34)	128 (31)	0.412
Ischemic lesions					
No lesions	119 (86)	111 (83)	116 (82)	346 (84)	0.723
Lacunes < 15 mm	16 (12)	16 (12)	22 (16)	54 (13)	0.536
Subcortical infarction ≥ 15 mm	2 (1)	0 (0)	0 (0)	2 (0.5)	0.137
Cortical infarction	4 (3)	9 (7)	7 (5)	20 (5)	0.334

Higher education was defined as at least 4 years of college or university. Cholesterol lowering medication was defined as the use of lipid modifying agents (C10). Antihypertensive medication was defined as the use of antihypertensives (C02), diuretics (C03), beta blocking agents (C07), calcium channel blockers (C08), or agents acting on the renin-angiotensin system (C09). Hypertension in the ACE 1950 Study first visit was defined as blood pressure ≥ 140/90 mmHg or the use of antihypertensive medication (C02, C03, C07, C08 or C09). Diabetes in the ACE 1950 Study first visit was defined as self-reported diabetes or increased levels of HbA1c (≥ 6.5%) or fasting blood glucose (≥ 7.0 mmol/l), or use of antidiabetic medication

^a^15 missing values

^b^17 missing values

^c^1 missing value

^d^1 missing value

^e^5 missing values

^f^3 missing values

^g^2 missing values

### Blood pressure and the presence of lacunes and severity of WMHs at age 70

Table 2 demonstrates the association between blood pressure categories at age 40 and the odds ratio of lacunes and severity of WMH at age 70.

**Table 2:** Associations between blood pressure at age 40 and cerebral small vessel disease at age 70 years, *n* = 414

		**Presence of lacunes**		**Fazekas scale**	
		**OR (95% CI)**		**OR (95% CI)**	
	**n (%)**	**Crude**^**a**^	**Adjusted**^**b**^	**Crude**^**a**^	**Adjusted**^**b**^
Age 40 Program					
Systolic BP groups					
< 120 mm Hg	139 (34)	1 (ref)	1 (ref)	1 (ref)	1 (ref)
130–139 mm Hg	134 (32)	1.02 (0.49–2.15)	0.80 (0.35–1.84)	1.22 (0.77–1.95)	1.15 (0.68–1.93)
≥ 140 mm Hg	141 (34)	1.38 (0.69–2.77)	1.05 (0.46–2.38)	1.32 (0.83–2.11)	1.29 (0.75–2.21)
Diastolic BP groups					
< 70 mm Hg	75 (18)	1 (ref)	1 (ref)	1 (ref)	1 (ref)
70–79 mm Hg	144 (35)	1.67 (0.63–4.42)	1.50 (0.55–4.14)	1.23 (0.71–2.13)	1.27 (0.71–2.27)
80–89 mm Hg	122 (30)	1.89 (0.71–5.07)	1.55 (0.52–4.57)	1.75 (0.99–3.08)	1.91 (1.01–3.62)
≥ 90 mm Hg	73 (18)	2.55 (0.91–7.18)	2.22 (0.73–6.80)	1.89 (1.00–3.56)	2.11 (1.06–4.19)

^a^Adjusted for age at brain magnetic resonance imaging (MRI)

^b^Adjusted for sex, education (ACE 1950), smoking at age 40, total cholesterol at age 40, physical activity at age 40, diabetes at age 40, and age at brain MRI

High elevated systolic blood pressure (130–139 mmHg) and hypertension (≥ 140 mmHg) at age 40 were not significantly associated with the presence of lacunes or the Fazekas scale, compared to non-elevated systolic blood pressure (< 120 mmHg) neither in the crude nor in the adjusted analyses.

For diastolic blood pressure at age 40, no significant association was found with lacunes in the crude or adjusted analysis. However, diastolic hypertension (≥ 90 mmHg) was associated with Fazekas scale in the crude analysis (OR [95% CI] 1.89 [1.00–3.56]. After adjusting for sex, education, total cholesterol, diabetes, smoking, physical activity, and age at MRI, both high elevated diastolic blood pressure (80–89 mmHg, OR 1.91 [1.01–3.62]) and diastolic hypertension (≥ 90 mmHg, OR 2.11 [1.06–4.19]) were significantly associated with the Fazekas scale, compared to the reference group (< 70 mmHg).

In sensitivity analysis, after exclusion of physical activity as a covariate, high elevated diastolic blood pressure (80–89 mmHg) and diastolic hypertension (≥ 90 mmHg) were no longer significantly associated with Fazekas scale (Supplemental Table S4).

No significant associations between systolic or diastolic blood pressure in the mid-60s and the presence of lacunes and Fazekas scale were found (Supplemental Table S5).

### Blood pressure trajectories and the presence of lacunes and severity of WMHs

**Fig. 3:**
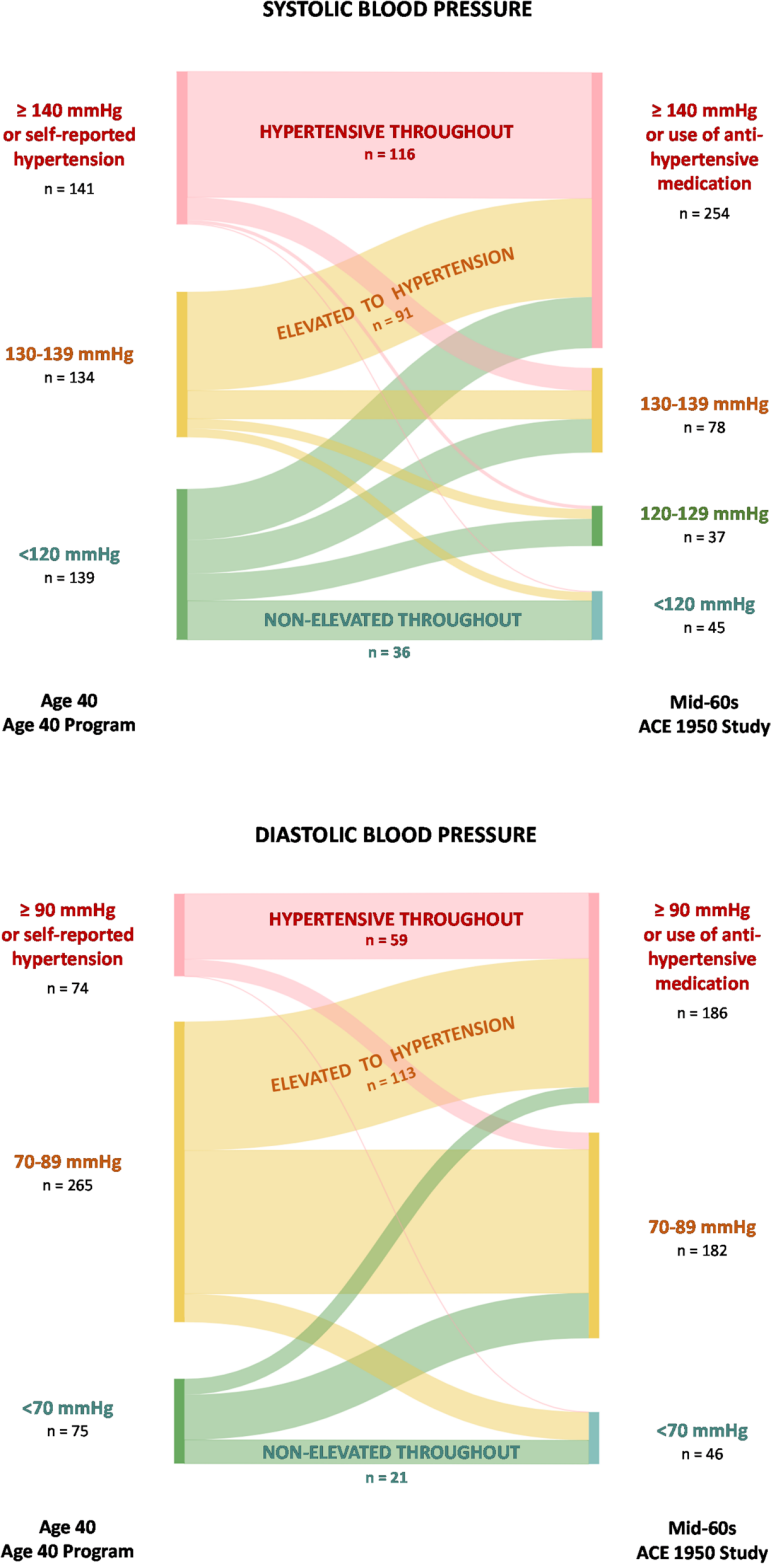
Blood pressure trajectories between age 40 and the mid-60s. Footnote: Figure developed by the authors using SankeyMATIC

Between age 40 and the mid-60s, 91 (37%) participants had a trajectory of high elevated systolic blood pressure (130–139 mmHg) to systolic hypertension, and 116 (48%) had systolic hypertension throughout, while 113 (59%) had a trajectory of elevated diastolic blood pressure (70–89 mmHg) to diastolic hypertension, and 59 (31%) had diastolic hypertension throughout (Fig. 3 ). Table 3 shows the association between blood pressure trajectories between age 40 and the mid-60s, and the presence of lacunes and Fazekas scale.

**Table 3:** Associations between blood pressure trajectories including antihypertensive treatment between age 40 and the mid-60s and neuroimaging features of cerebral small vessel disease at age 70 years

		**Lacunes**			**Fazekas scale**				
	**Total (%)**	**n**^**a**^** (%)**	**OR**^**b**^** (95% CI)**	**aOR**^**c**^** (95% CI)**	**Fazekas scale** ** ≥ 2 (%)**	**OR**^**b**^** (95% CI)**	**aOR**^**c**^** (95% CI)**		
Systolic blood pressure trajectories								Diastolic BP at age 40, mmHg, mean ± SD	Diastolic BP in mid-60s, mmHg, mean ± SD
Non-elevated throughout	36 (15)	2 (6)	1 (ref)	1 (ref)	7 (19)	1 (ref)	1 (ref)	69.1 ± 5.2	67.1 ± 7.6
High elevated to hypertension	91 (37)	12 (13)	2.46 (0.52–11.71)	1.41 (0.25–7.79)	33 (36)	2.65 (1.21–5.82)	3.00 (1.24–7.24)	83.1 ± 7.5	80.8 ± 9.5
Hypertension throughout	116 (48)	20 (17)	3.39 (0.75–15.40)	1.75 (0.32–9.55)	39 (34)	2.38 (1.11–5.13)	2.42 (1.01–5.78)	87.9 ± 8.3	83.3 ± 9.6
Total	243 (100)	34 (14)	-	-	79 (33)	-	-	83.3 ± 9.9	80.0 ± 10.8
Diastolic blood pressure trajectories								Systolic BP at age 40, mmHg, mean ± SD	Systolic BP in mid-60s, mmHg, mean ± SD
Non-elevated throughout	21 (11)	2 (10)	1 (ref)	1 (ref)	4 (19)	1 (ref)	1 (ref)	111.6 ± 11.2	119.3 ± 11.2
Elevated to hypertension	113 (59)	20 (18)	2.10 (0.45–9.84)	0.65 (0.10–4.20)	44 (39)	2.23 (0.90–5.52)	2.71 (0.90–8.24)	136.8 ± 10.6	144.2 ± 18.7
Hypertension throughout	59 (31)	11 (19)	2.32 (0.46–11.63)	1.45 (0.21–9.89)	25 (42)	2.37 (0.90–6.28)	3.12 (0.97–10.06)	147.3 ± 10.4	146.4 ± 17.1
Total	193 (100)	33 (17)	-	-	73 (38)	-	-	137.3 ± 14.6	142.2 ± 19.3

^a^Number of individuals with lacunes

^b^Adjusted for age at brain magnetic resonance imaging (MRI)

^c^Adjusted for sex, education (ACE 1950), smoking (ACE 1950), hypercholesterolemia (ACE 1950), physical activity (ACE 1950), history of stroke (ACE 1950), diabetes (ACE 1950), and age at brain MRI. *Systolic blood pressure: Non-elevated throughout,* no more than 120 mmHg in the Age 40 Program and in the ACE 1950 Study first visit; *High elevated to hypertension*, blood pressure of 130–139 mmHg in the Age 40 Program and at least 140 mmHg or use of antihypertensive medication in the ACE 1950 Study first visit; *Hypertension throughout*, blood pressure of at least 140 mmHg or self-reported hypertension in the Age 40 Program and a systolic blood pressure of at least 140 mmHg or the use of antihypertensive medication in the ACE 1950 Study first visit. *Diastolic blood pressure: Non-elevated throughout*, blood pressure of no more than 70 mmHg in the Age 40 Program and in the ACE 1950 Study first visit; *Elevated to Hypertension*, blood pressure of 70–89 mmHg in the Age 40 Program and at least 90 mmHg or the use of antihypertensive medication in the ACE 1950 Study first visit; *Hypertension throughout*, blood pressure of at least 90 mmHg or self-reported hypertension in the Age 40 Program and a diastolic blood pressure of at least 90 mmHg or the use of antihypertensive medication in the ACE 1950 Study first visit

Using non-elevated systolic blood pressure (< 120 mmHg) throughout as the reference group, the trajectories of high elevated systolic blood pressure to systolic hypertension and systolic hypertension throughout were associated with Fazekas scale in both unadjusted (OR [95% CI], 2.65 [1.21–5.82] and 2.38 [1.11–5.13]) and adjusted analysis (OR [95% CI], 3.00 [1.24–7.24] and 2.42 [1.01–5.78], respectively), but not with the presence of lacunes.

Participants with sustained diastolic hypertension had an adjusted OR (95% CI) of 3.12 (0.97–10.06) for a higher Fazekas scale compared to those with non-elevated diastolic blood pressure (< 70 mmHg) throughout. No significant association was found for diastolic blood pressure progressing from elevated to hypertensive (adjusted OR [95% CI], 2.71 [0.90–8.24]). No association was found between diastolic blood pressure trajectories and the presence of lacunes.

In the sensitivity analysis, investigating the association between systolic and diastolic blood pressure trajectories and the presence of lacunes and Fazekas scale, regardless of antihypertensive use, only the progression of high elevated systolic blood pressure (130–139 mmHg) to systolic hypertension (≥ 140 mmHg) remained significant (adjusted OR [95% CI] 3.70 [1.40–9.73]), while the crude association between sustained diastolic hypertension and lacunes became significant (OR 6.80 [1.11–41.63], Supplemental Table S6). When excluding physical activity as a covariate (Supplemental Table S7), the trajectory of sustained systolic hypertension was no longer significantly associated with Fazekas scale.

## Discussion

In this longitudinal study from the general population in Norway, we found that, in contrast to elevated and hypertensive systolic blood pressure, high elevated diastolic blood pressure (80–89 mmHg) and diastolic hypertension (> 90 mmHg) at age 40 were associated with the severity of WMH on brain MRI around 30 years later. When exploring blood pressure trajectories, sustained systolic hypertension, and increased systolic blood pressure over the course of midlife, were associated with more severe degrees of WMHs. We did not find any significant association between blood pressure and the presence of lacunes.

### Systolic and diastolic blood pressure at age 40 and neuroimaging features of cSVD

The significant associations between blood pressure at age 40 and WMH were observed only for diastolic blood pressure. Individuals with diastolic blood pressure in the categories of 80–89 mmHg and ≥ 90 mmHg at age 40 had approximately two-fold adjusted odds of having a higher Fazekas scale at age 70 compared to the reference group. This finding highlights the importance of diastolic blood pressure on WMH development and aligns with results from previous observational studies [29–33]. In contrast to our study, previous investigations have included populations aged 50 and older, with a subsequent shorter follow-up time. These findings together with ours, and recent literature [15], support the notion that the *early* years of midlife (ages 40–50) may be a particularly sensitive time window for the negative effects of elevated blood pressure on subsequent WMH development, with notable impact from diastolic blood pressure. Our findings may support the current guidelines recommending hypertension screening already from age 40 [19], but importantly, screening for hypertension should be based on multiple criteria, and the evidence supporting pharmacological treatment in this age group is limited [34–36].

### Blood pressure trajectories and neuroimaging features of cSVD

The trajectories of high elevated systolic blood pressure to systolic hypertension and systolic hypertension throughout in the mid-60s were positively associated with WMH, aligning with the prevailing view that elevated systolic blood pressure during (*late*-) midlife is a prominent risk factor for WMH burden in later life [18, 37, 38]. The association between sustained diastolic hypertension and WMH trended towards significance, while progression from elevated diastolic blood pressure to diastolic hypertension was non-significant. Previous studies have reported a J-shaped association between long-term change in diastolic blood pressure and severity of WMHs [39, 40]. However, our study could not confirm a J-shaped association because decreasing diastolic blood pressure trajectories were not investigated. Overall, our findings suggest that both high diastolic and sustained systolic blood pressure are risk factors for subsequent WMH development, but their contributions may vary across the lifespan.

### No association between blood pressure and the presence of lacunes

We did not find significant associations between blood pressure and lacunes. This may be explained by the heterogeneity and likely multifactorial etiology of lacunes [10]. Lacunes are highly correlated with age in healthy elderly individuals, with prevalence increasing from around 8% at age 60 to 35% at age 90 [2]. Previous research has been limited by a lack of consistent terminology on entities such as “lacunes”, “incidental, silent or covert brain infarcts”, and “asymptomatic lacunar infarction”, hindering effective knowledge synthesis and clear guidelines [41]. Thus, the clinical relevance of these lesions remains uncertain and debated [42–45].

### Strengths and limitations

There are several strengths to our study. First, blood pressure was measured several times and according to a predefined study protocol both in the Age 40 Program and the ACE 1950 Study first visit. The use of repeated blood pressure measurements provides insights into the effects of hypertension exposure throughout adulthood. The very long follow-up of around 30 years is a further advantage compared to previous studies. The ACE 1950 Study is a well-described population, and our results add to the knowledge of cardiovascular risk factors in this population. Blood pressure trajectories were defined in a pragmatic and clinically relevant way, applying categories and thresholds from recently published blood pressure guidelines. Moreover, all brain MRIs were performed according to the same study protocol, and all scans were interpreted by experienced radiologists [46]. Finally, the inclusion of participants born in the same year limited confounding effects from age, which is strongly correlated to WMH burden and the presence of lacunes.

There are also limitations to acknowledge. We used a single-time point measurement of blood pressure as the main exposure, while more frequent measurements or the use of ambulatory blood pressure would have reduced measurement error and bias from confounders such as white-coat hypertension. These effects were probably attenuated by serial measurements after a predefined period of rest, but we cannot exclude residual confounding. Further, participants were selected into the present study after participation in both the Age 40 Program and the ACE 1950 Study first visit and then invited to perform brain MRI based on the strata < 120 mmHg, 130–139 mmHg and ≥ 140 mmHg of systolic blood pressure at age 40, excluding the group with SBP 120–129 mmHg. This increases the risk of selection bias. Also, there is risk of survival bias, as deceased participants between the Age 40 Program and the ACE 1950 Study first visit would not be eligible for inclusion into the present study. Analyses of blood pressure trajectories were performed on a subset of the study population, and these findings need to be replicated in larger cohorts. We did not analyse other neuroimaging features of cSVD, and we did not measure the size or localisation of lacunes and WMHs. Lastly, there were slight differences in blood pressure measurement protocols between the Age 40 Program and the ACE 1950 Study first visit, and this may have influenced the comparability of blood pressure values. Our study may be clinically useful because it demonstrates that the association between elevated diastolic and systolic blood pressure and brain health likely varies across the lifespan, with diastolic hypertension having a greater effect earlier in life and systolic hypertension becoming more significant in later years.

## Conclusions

In this study of 414 individuals from the general Norwegian population, we found that elevated diastolic blood pressure (80–89 mmHg) and diastolic hypertension (> 90 mmHg) at age 40 were independently associated with the severity of WMHs three decades later, and that sustained high systolic blood pressure or increased systolic blood pressure trajectories over the course of midlife were associated with WMH severity. The presence of lacunes did not seem to be associated with blood pressure.

## Supplementary Information

Supplementary Material 1.

## Data Availability

The datasets generated and analysed during the current study are not publicly available because the Data Protection Authority approval and patient consent do not allow for such publication, but data are available from the corresponding author on reasonable request. More information is available on the study website (http://www.ace1950.no).

## Abbreviations

ACE 1950 Study: Akershus Cardiac Examination 1950 Study
CI: Confidence interval
DBP: Diastolic blood pressure
HR: Hazard ratio
MRI: Magnetic resonance imaging
OR: Odds ratio
SD: Standard deviation
SPB: Systolic blood pressure
STROBE: Strengthening The Reporting of OBservational studies in Epidemiology
cSVD: Cerebral small vessel disease
WMH: White matter hyperintensity
