# Supplementary material for: Blood pressure at age 40 and key features of cerebral small vessel disease at age 70: data from the ACE 1950 Study
Source: BMC Cardiovasc Disord. 2025 Oct 9;25:724. doi: 10.1186/s12872-025-05140-6 (PMC12509344; doi:10.1186/s12872-025-05140-6)
Supplement: Supplementary file 1 — Supplementary Material 1. [file 12872_2025_5140_MOESM1_ESM.docx]

**SUPPLEMENTARY MATERIAL**

**Blood Pressure at Age 40 and Key Features of Cerebral Small Vessel
Disease at Age 70: Data from the ACE 1950 Study**

Marte M. Walle-Hansen, MD^a,b^, Guri Hagberg, MD PhD^a,b,c^, Marius Myrstad, MD PhD^a,b,d^, Trygve Berge, MD PhD^a,d^, Thea Vigen, MD PhD^e^, Hege Ihle-Hansen, MD PhD^a,k^, Bente Thommessen, MD PhD^e^, Inger Ariansen, MD PhD^f^, Magnus N. Lyngbakken, MD PhD^g,h^, Helge Røsjø, MD PhD^h,i^, Ole M. Rønning, MD PhD^b,e^, Mona K Beyer MD PhD^b,j^ Arnljot Tveit, MD PhD^a,b^ and Håkon Ihle-Hansen, MD PhD^a,d^

1. Department of Medical Research, Bærum Hospital, Vestre Viken Hospital Trust, Gjettum, Norway
2. Institute of Clinical Medicine, University of Oslo, Oslo, Norway
3. Stroke unit, Department of Neurology, Oslo University Hospital, Ullevål, Norway
4. Department of Internal Medicine, Bærum Hospital, Vestre Viken Hospital Trust, Drammen, Norway
5. Department of Neurology, Division of Medicine, Akershus University Hospital, Lørenskog, Norway
6. Department of Chronic Diseases, Norwegian Institute of Public Health, Oslo, Norway
7. Department of Cardiology, Division of Medicine, Akershus University Hospital, Lørenskog, Norway
8. K.G. Jebsen Center for Cardiac Biomarkers, Institute of Clinical Medicine, University of Oslo, Norway
9. Akershus Clinical Research Center (ACR), Division of Research and Innovation, Akershus University Hospital, Lørenskog, Norway
10. Division of Radiology and Nuclear Medicine, Oslo University Hospital, Oslo, Norway
11. Department of Acute Medicine, Oslo University Hospital, Oslo, Norway

**Corresponding Author:** Marte Meyer Walle-Hansen, MD
Department of Medical Research, Baerum Hospital
Sogneprest Munthe-Kaas vei 100, 1346 Gjettum, Norway
Email: [marte.meyer.walle-hansen@vestreviken.no](mailto:marte.meyer.walle-hansen@vestreviken.no)
Telephone: (00 47) 99 29 40 01

**Contents:**

**Supplemental Table S1**: Description of Covariate Measurements

**Supplemental Table S2:** Population Characteristics by Sex, n=414

**Supplemental Table S3:** Characteristics of Participants from the ACE 1950 Study With and Without Brain Magnetic Resonance Imaging, n = 3706 **Supplemental Table S4:** Associations Between Blood Pressure at Age 40 and Cerebral Small Vessel Disease at Age 70 Years, Without Adjustment of Physical Activity, n=414 **Supplemental Table S5:** Blood Pressure in the Mid-60s and Cerebral Small Vessel Disease at Age 70, n=414 **Supplemental Table S6**: Blood Pressure Trajectories Excluding Antihypertensive Treatment and Cerebral Small Vessel Disease at Age 70 **Supplemental Table S7:** Associations Between Blood Pressure Trajectories Including Antihypertensive Treatment Between Age 40 and the Mid-60s and Neuroimaging Features of Cerebral Small Vessel Disease at Age 70 Years, Without Adjustment of Physical Activity

**Supplemental Table S1: Description of Covariate Measurements**

| **Covariate** | **Description** |
| --- | --- |
| Total cholesterol  (Age 40 Program) | mmol per liter (mmol/L) |
| Daily smoking  (Age 40 Program) | Self-reported by questionnaire, with a yes or no response to the following questions:  “*Do you smoke cigarettes daily?*” and “*If you are not a daily smoker now, have you been a daily smoker previously?*” |
| Physical activity  (Age 40 Program) | Self-reported by questionnaire using the Saltin-Grimby Physical Activity Level Scale, with four response categories:  (1) Reading, watching TV, or other sedentary activity  (2) Walking, cycling, or other forms of exercise at least 4 h per week  (3) Participation in recreational sports, heavy gardening etc. at least 4 h per week  (4) Participation in hard training or sports competitions regularly, several times a week |
| History of diabetes  (Age 40 Program) | Self-reported by questionnaire with a yes or no response to the following question:  “*Do you currently have, or have you previously had diabetes?*” |
| Body mass index  (Age 40 Program) | Weight in kilograms divided by the square of height in meters |
| Obesity  (Age 40 Program) | Body mass index of at least 30 kg/m^2^ |
| Education  (ACE 1950 Study) | Self-reported by questionnaire in the ACE 1950 Study first visit by response to the following question:  “*What is the highest level of education you have completed?*”  (1) Less than 7 years of primary school  (2) Primary school 7-10 years  (3) Vocational upper secondary school  (4) General upper secondary school  (5) College or university, less than 4 years  (6) College or university, 4 years or more |
| Smoking  (ACE 1950 Study) | Self-reported by questionnaire in the ACE 1950 Study first visit and categorized into the following groups:  (1) Never smoked  (2) Daily smoker  (3) Previous smoker  (4) Occasional smoker |
| Hypercholesterolemia (ACE 1950 Study) | The self-reported use of statins or elevated total cholesterol (≥6.2 mmol/l) or elevated low-density lipoprotein (LDL)-cholesterol (≥4.1 mmol/l) |
| History of stroke  (ACE 1950 Study) | Self-reported by questionnaire with a yes or no response to the following question:  *“Have you previously had a stroke (blood clot or bleeding in the brain) or a transient ischemic attack (meaning that the symptoms resolved completely within 24 hours)?”* |
| Diabetes  (ACE 1950 Study) | Self-reported history of diabetes mellitus or increased levels of HbA1c (≥6.5%) or fasting blood glucose (≥7.0 mmol/l) or use of antidiabetic medication |
| Physical activity  (ACE 1950 Study) | Self-reported by questionnaire with response to the following question “*How often do you exercise? Give an average*”, with the following response categories:  (1) Never  (2) Less than once a week  (3) Once a week  (4) 2-3 times a week  (5) Almost every day |

**Supplemental Table S2: Population Characteristics by Sex, n=414**

|  | **Men** | **Women** | **Total** | **P value** |
| --- | --- | --- | --- | --- |
| **N (%)** | 247 (60) | 167 (40) | 414 (100) |  |
| **Age 40 Program** |  |  |  |  |
| Age, years, mean ± SD | 40.1 ± 0.3 | 40.1 ± 0.4 | 40.1 ± 0.3 | 0.537 |
| Body mass index^a^, kg/m^2^, mean ± SD | 25.4 ± 2.7 | 23.5 ± 3.2 | 24.6 ± 3.0 | <0.01 |
| Obesity | 24 (10) | 12 (7) | 36 (9) | 0.370 |
| Blood pressure, mm Hg, mean ± SD |  |  |  |  |
| Systolic | 137.5 ± 12.1 | 122.2 ± 15.4 | 131.3 ± 15.5 | <0.01 |
| Diastolic | 82.6 ± 9.1 | 75.7 ± 10.2 | 79.8 ± 10.1 | <0.01 |
| Non-Caucasian | 4 (2) | 3 (2) | 7 (2) | 0.891 |
| Physical activity |  |  |  | <0.01 |
| Sedentary activity | 41 (16) | 29 (17) | 70 (17) |  |
| Walking or cycling ≥ 4 h/week | 133 (54) | 118 (71) | 251 (61) |  |
| Recreational sports ≥ 4 h/week | 64 (26) | 19 (11) | 83 (20) |  |
| Hard training several times/week | 9 (4) | 1 (1) | 10 (2) |  |
| Smoking^b^ |  |  |  | 0.818 |
| Never | 97 (41) | 65 (40) | 162 (41) |  |
| Current or previous | 138 (59) | 97 (60) | 235 (59) |  |
| Cardiovascular comorbidity |  |  |  |  |
| Myocardial infarction or angina | 2 (1) | 0 (0) | 2 (0.5) | 0.244 |
| Hypertension^c^ | 5 (2) | 2 (1) | 7 (2) | 0.519 |
| Diabetes | 0 (0) | 0 (0) | 0 (0) | - |
| Biomarkers, mean ± SD |  |  |  |  |
| Cholesterol, mmol/L | 5.7 ± 1.1 | 5.2 ± 1.0 | 5.5 ± 1.1 | <0.01 |
| Triglycerides, mmol/L | 2.2 ± 1.2 | 1.2 ± 0.8 | 1.8 ± 1.1 | <0.01 |
| **ACE 1950 Study** |  |  |  |  |
| Age, years, mean ± SD | 64.0 ± 0.6 | 63.9 ± 0.7 | 63.9 ± 0.7 | 0.132 |
| Body mass index, kg/m^2^, mean ± SD | 27.3 ± 3.3 | 26.8 ± 4.6 | 27.1 ± 3.9 | 0.146 |
| Higher education^d^ | 123 (50) | 75 (45) | 198 (48) | 0.310 |
| Blood pressure, mm Hg, mean ± SD |  |  |  |  |
| Systolic | 139.7 ± 16.7 | 136.5 ± 18.0 | 138.4 ± 17.3 | 0.066 |
| Diastolic | 81.0 ± 9.0 | 74.2 ± 9.6 | 78.3 ± 9.8 | <0.01 |
| Physically active at least 30 min/day^e^ | 203 (83) | 149 (90) | 352 (86) | 0.042 |
| Smoking^f^ |  |  |  | 0.388 |
| Never | 87 (35) | 58 (35) | 145 (35) |  |
| Daily | 26 (11) | 25 (15) | 51 (13) |  |
| Previous | 126 (51) | 80 (49) | 206 (50) |  |
| Occasional | 7 (3) | 2 (1) | 9 (2) |  |
| Medication use |  |  |  |  |
| Cholesterol lowering medication | 70 (28) | 43 (26) | 113 (27) | 0.561 |
| Antihypertensive medication | 97 (39) | 56 (34) | 153 (37) | 0.235 |
| Antidiabetic medication | 20 (8) | 3 (2) | 23 (6) | <0.01 |
| Antiplatelet medication | 53 (21) | 19 (11) | 72 (17) | <0.01 |
| Anticoagulative medication | 14 (6) | 3 (2) | 17 (4) | 0.051 |
| Cardiovascular comorbidity |  |  |  |  |
| Hypertension | 162 (66) | 92 (55) | 254 (61) | <0.01 |
| Cardiac disease | 36 (15) | 8 (5) | 44 (11) | <0.01 |
| Diabetes | 26 (11) | 10 (6) | 36 (9) | 0.108 |
| Obesity | 53 (21) | 39 (23) | 92 (22) | 0.649 |

**Supplemental Table S2 continued: Population Characteristics by Sex, n=414**

|  | **Men** | **Women** | **Total** | **P value** |
| --- | --- | --- | --- | --- |
| **N (%)** | 247 (60) | 167 (40) | 414 (100) |  |
| **Brain MRI findings** |  |  |  |  |
| Age at MRI, years, mean ± SD | 70.1 ± 2.3 | 70.3 ± 2.2 | 70.2 ± 2.3 | 0.214 |
| **Fazekas scale^g^** |  |  |  | 0.586 |
| 0: No hyperintensities | 26 (11) | 15 (9) | 41 (10) |  |
| 1: Punctate foci | 140 (57) | 103 (62) | 243 (59) |  |
| 2: Beginning confluence | 45 (18) | 31 (19) | 76 (18) |  |
| 3: Large confluent areas | 35 (14) | 17 (10) | 52 (13) |  |
| Fazekas scale ≥2 | 80 (33) | 48 (29) | 128 (31) | 0.438 |
| **Ischemic lesions** |  |  |  |  |
| No lesions | 197 (80) | 149 (89) | 346 (84) | 0.011 |
| Lacunes < 15 mm | 37 (15) | 17 (10) | 54 (13) | 0.155 |
| Subcortical infarction >15 mm | 2 (1) | 0 (0) | 2 (0.5) | 0.244 |
| Cortical infarction | 19 (8) | 1 (1) | 20 (5) | <0.01 |

Footnote: ^a^ 15 missing values, ^b^ 17 missing values, ^c^ 1 missing value, ^d^ 1 missing value, ^e^ 5 missing values, ^f^ 3 missing values,
^g^ 2 missing values. Higher education was defined as at least 4 years of college or university. Cholesterol lowering medication was defined as the use of lipid modifying agents (C10). Antihypertensive medication was defined as the use of anihypertensives (C02), diuretics (C03), beta blocking agents (C07), calcium channel blockers (C08), or agents acting on the renin-angiotensin system (C09). Antidiabetic medication was defined as use of insulins and analogues (A10A), and blood glucose lowering drugs excluding insulins (A10B). Antiplatelet medication was defined as the use of platelet aggregation inhibitors excluding heparin (B01AC). Anticoagulative medication was defined as the use of vitamin K antagonists (B01AA), dabigatran etexilate (B01AE07), rivaroxaban (B01AF01), or apixaban (B01AF02). Hypertension in the ACE 1950 Study first visit was defined as blood pressure ≥140/90 mmHg or the use of antihypertensive medication (C02, C03, C07, C08 or C09). Cardiac disease in the ACE 1950 Study first visit was defined as a history of atrial fibrillation, coronary heart disease, angina pectoris, or heart failure. Diabetes in the ACE 1950 Study first visit was defined as self-reported diabetes or increased levels of HbA1c (≥6.5%) or fasting blood glucose (≥7.0 mmol/l), or use of antidiabetic medication.

**Supplemental Table S3: Characteristics of Participants from the ACE 1950 Study With and Without Brain Magnetic Resonance Imaging, n = 3706**

|  | **Participants without brain MRI**  **n = 3292** | **Participants with**  **brain MRI**  **n = 414** | **P value** |
| --- | --- | --- | --- |
| Age, years, mean ± SD | 63.9±0.6 | 63.9±0.7 | 0.870 |
| Body mass index, kg/m^2^, mean ± SD | 27.2±4.5 | 27.1±3.9 | 0.838 |
| Higher education^a^, n (%) | 1515 (46) | 198 (48) | 0.494 |
| Blood pressure, mm Hg, mean ± SD |  |  |  |
| Systolic^b^ | 137.8±18.9 | 138.4±17.3 | 0.554 |
| Diastolic^c^ | 76.8±10.1 | 78.3±9.8 | <0.01 |
| Physically active at least 30 min/day^d^, n (%) | 2739 (84) | 352 (86) | 0.396 |
| Smoking^e^, n (%) |  |  | 0.077 |
| Never | 1103 (34) | 145 (35) |  |
| Daily | 481 (15) | 51 (12) |  |
| Previous | 1535 (47) | 206 (50) |  |
| Occasional | 146 (4) | 9 (2) |  |
| Medication use, n (%) |  |  |  |
| Cholesterol lowering medication | 857 (26) | 113 (27) | 0.582 |
| Antihypertensive medication | 1193 (36) | 153 (37) | 0.775 |
| Antidiabetic medication | 179 (5) | 23 (6) | 0.921 |
| Antiplatelet medication | 563 (17) | 72 (17) | 0.883 |
| Anticoagulative medication | 107 (3) | 17 (4) | 0.361 |
| Cardiovascular comorbidity, n (%) |  |  |  |
| Hypertension^f^ | 2041 (62) | 256 (62) | 0.943 |
| Cardiac disease | 420 (13) | 44 (11) | 0.217 |
| Diabetes^g^ | 281 (9) | 36 (9) | 0.916 |
| Obesity | 747 (23) | 92 (22) | 0.830 |

Footnote: ^a^ 11 missing values, ^b^ 4 missing values, ^c^ 4 missing values, ^d^ 54 missing values, ^e^ 30 missing values, ^f^ 1 missing value, ^g^ 2 missing values. MRI, magnetic brain resonance imaging. Cholesterol lowering medication was defined as the use of lipid modifying agents (C10). Antihypertensive medication was defined as the use of anihypertensives (C02), diuretics (C03), beta blocking agents (C07), calcium channel blockers (C08), or agents acting on the renin-angiotensin system (C09). Antidiabetic medication was defined as use of insulins and analogues (A10A), and blood glucose lowering drugs excluding insulins (A10B). Antiplatelet medication was defined as the use of platelet aggregation inhibitors excluding heparin (B01AC). Anticoagulative medication was defined as the use of vitamin K antagonists (B01AA), dabigatran etexilate (B01AE07), rivaroxaban (B01AF01), or apixaban (B01AF02). Hypertension in the ACE 1950 Study first visit was defined as blood pressure ≥140/90 mmHg or the use of antihypertensive medication (C02, C03, C07, C08 or C09). Cardiac disease in the ACE 1950 Study first visit was defined as a history of atrial fibrillation, coronary heart disease, angina pectoris, or heart failure. Diabetes in the ACE 1950 Study first visit was defined as self-reported diabetes or increased levels of HbA1c (≥6.5%) or fasting blood glucose (≥7.0 mmol/l), or use of antidiabetic medication. Obesity was defined as BMI ≥30 kg/m^2^.

**Supplemental Table S4: Associations Between Blood Pressure at Age 40 and Cerebral Small Vessel Disease at Age 70 Years, Without Adjustment of Physical Activity, n=414**

|  |  | **Presence of lacunes** | | **Fazekas scale** | |
| --- | --- | --- | --- | --- | --- |
|  |  | **OR (95% CI)** | | **OR (95% CI)** | |
|  | **n (%)** | **Crude^a^** | **Adjusted^b^** | **Crude^a^** | **Adjusted^b^** |
| **Age 40 Program** |  |  |  |  |  |
| **Systolic BP groups** |  |  |  |  |  |
| <120 mm Hg | 139 (34) | 1 (ref) | 1 (ref) | 1 (ref) | 1 (ref) |
| 130-139 mm Hg | 134 (32) | 1.02 (0.49-2.15) | 0.80 (0.35-1.83) | 1.22 (0.77-1.95) | 1.17 (0.70-1.97) |
| ≥140 mm Hg | 141 (34) | 1.38 (0.69-2.77) | 1.03 (0.46-2.32) | 1.32 (0.83-2.11) | 1.28 (0.75-2.19) |
| **Diastolic BP groups** |  |  |  |  |  |
| <70 mm Hg | 75 (18) | 1 (ref) | 1 (ref) | 1 (ref) | 1 (ref) |
| 70-79 mm Hg | 144 (35) | 1.67 (0.63-4.42) | 1.53 (0.56-4.21) | 1.23 (0.71-2.13) | 1.23 (0.69-2.19) |
| 80-89 mm Hg | 122 (30) | 1.89 (0.71-5.07) | 1.54 (0.52-4.53) | 1.75 (0.99-3.08) | 1.82 (0.96-3.42) |
| ≥90 mm Hg | 73 (18) | 2.55 (0.91-7.18) | 2.20 (0.72-6.71) | 1.89 (1.00-3.56) | 1.97 (0.99-3.88) |

^a^ Adjusted for age at brain magnetic resonance imaging (MRI); ^b^ Adjusted for sex, education (ACE 1950), smoking at age 40, total cholesterol at age 40, diabetes at age 40, and age at brain MRI.

**Supplemental Table S5: Blood Pressure in the Mid-60s and Cerebral Small Vessel Disease at Age 70, n=414**

|  |  | **Presence of lacunes** | | **Fazekas scale** | |
| --- | --- | --- | --- | --- | --- |
|  |  | **OR (95% CI)** | | **OR (95% CI)** | |
|  | **n (%)** | **Crude^a^** | **Adjusted^b^** | **Crude^a^** | **Adjusted^b^** |
| **ACE 1950 Study** |  |  |  |  |  |
| **Systolic BP groups** |  |  |  |  |  |
| <120 mm Hg | 55 (13) | 1 (ref) | 1 (ref) | 1 (ref) | 1 (ref) |
| 120-129 mm Hg | 67 (16) | 1.62 (0.55-4.72) | 1.52 (0.48-4.89) | 1.46 (0.73-2.93) | 1.37 (0.65-2.90) |
| 130-139 mm Hg | 114 (28) | 0.96 (0.34-2.71) | 0.68 (0.22-2.09) | 1.46 (0.77-2.78) | 1.29 (0.65-2.58) |
| ≥140 mm Hg | 178 (43) | 1.29 (0.50-3.33) | 0.79 (0.23-2.70) | 1.73 (0.95-3.17) | 1.17 (0.54-2.57) |
| **Diastolic BP groups** |  |  |  |  |  |
| <70 mm Hg | 67 (16) | 1 (ref) | 1 (ref) | 1 (ref) | 1 (ref) |
| 70-79 mm Hg | 178 (43) | 1.32 (0.54-3.25) | 1.18 (0.44-3.18) | 1.30 (0.75-2.24) | 1.26 (0.69-2.28) |
| 80-89 mm Hg | 114 (28) | 1.07 (0.40-2.87) | 0.74 (0.23-2.33) | 1.04 (0.57-1.90) | 0.75 (0.38-1.49) |
| ≥90 mm Hg | 55 (13) | 2.27 (0.82-6.27) | 1.80 (0.52-6.28) | 1.87 (0.93-3.77) | 1.28 (0.56-2.94) |

^a^ Adjusted for age at brain magnetic resonance imaging (MRI); ^b^ Adjusted for sex, education (ACE 1950), smoking (ACE 1950), hypercholesterolemia (ACE 1950), hypertension or use of antihypertensive medication (ACE 1950), stroke history (ACE 1950), physical activity (ACE 1950), diabetes (ACE 1950), and age at brain MRI.

**Supplemental Table S6:** **Blood Pressure Trajectories Excluding Antihypertensive Treatment and Cerebral Small Vessel Disease at Age 70**

|  |  |  | **Lacunes (logistic regression)** | | |  | **Fazekas scale (ordinal regression)** | | |  |  |  |
| --- | --- | --- | --- | --- | --- | --- | --- | --- | --- | --- | --- | --- |
|  | **Total (%)** |  | **n^a^ (%)** | **OR^b^ (95% CI)** | **aOR^c^ (95% CI)** |  | **Fazekas scale**  **≥2 (%)** | **OR^b^ (95% CI)** | **aOR^c^ (95% CI)** |  |  |  |
| **Systolic blood pressure trajectories** |  |  |  |  |  |  |  |  |  |  | Diastolic BP at age 40,  mmHg, mean±SD | Diastolic BP in mid-60s,  mmHg, mean±SD |
| Non-elevated throughout | 39 (22) |  | 2 (5) | 1 (ref) | 1 (ref) |  | 8 (21) | 1 (ref) | 1 (ref) |  | 69.3±5.3 | 67.2±7.3 |
| High elevated to hypertension | 64 (35) |  | 8 (13) | 2.52 (0.50-12.61) | 1.90 (0.28-12.70) |  | 25 (39) | 2.77 (1.24-6.18) | 3.70 (1.40-9.73) |  | 83.0±7.7 | 83.4±8.6 |
| Hypertension throughout | 79 (43) |  | 15 (19) | 4.14 (0.89-19.25) | 3.12 (0.48-20.10) |  | 25 (32) | 1.98 (0.91-4.30) | 1.84 (0.71-4.74) |  | 87.7±8.9 | 86.6±8.7 |
| Total | 182 (100) |  | 25 (14) | - | - |  | 58 (32) | - | - |  | 82.1±10.5 | 81.3±11.3 |
| **Diastolic blood pressure trajectories** |  |  |  |  |  |  |  |  |  |  | Systolic BP  at age 40,  mmHg, mean±SD | Systolic BP  in mid-60s,  mmHg, mean±SD |
| Non-elevated throughout | 25 (32) |  | 2 (8) | 1 (ref) | 1 (ref) |  | 6 (24) | 1 (ref) | 1 (ref) |  | 111.5±10.5 | 120.9±12.0 |
| Elevated to hypertension | 30 (38) |  | 5 (17) | 2.25 (0.38-13.36) | 0.36 (0.01-9.86) |  | 12 (40) | 1.96 (0.68-5.67) | 1.72 (0.31-9.63) |  | 136.0±10.6 | 161.3±14.4 |
| Hypertension throughout | 23 (30) |  | 7 (30) | 6.80 (1.11-41.63) | 6.11 (0.21-176.03) |  | 11 (48) | 2.12 (0.71-6.33) | 2.82 (0.48-16.78) |  | 145.3±9.8 | 158.2±15.8 |
| Total | 78 (100) |  | 14 (18) | - | - |  | 29 (37) | - | - |  | 130.9±17.3 | 147.4±23.1 |

^a^ Number of individuals with lacunes; ^b^ Adjusted for age at brain magnetic resonance imaging (MRI); ^c^ Adjusted for sex, education (ACE 1950), smoking (ACE 1950), hypercholesterolemia (ACE 1950), physical activity (ACE 1950), history of stroke (ACE 1950), diabetes (ACE 1950), and age at brain MRI. *Systolic blood pressure: Non-elevated throughout,* no more than 120 mmHg in the Age 40 Program and in the ACE 1950 Study first visit; *High elevated to hypertension*, blood pressure of 130-139 mmHg in the Age 40 Program and at least 140 mmHg in the ACE 1950 Study first visit; *Hypertension throughout*, blood pressure of at least 140 mmHg in the Age 40 Program and a systolic blood pressure of at least 140 mmHg in the ACE 1950 Study first visit. *Diastolic blood pressure: Non-elevated throughout*, blood pressure of no more than 70 mmHg in the Age 40 Program and in the ACE 1950 Study first visit; *Elevated to hypertension*, blood pressure of 70-89 mmHg in the Age 40 Program and at least 90 mmHg in the ACE 1950 Study first visit; *Hypertension throughout*, blood pressure of at least 90 mmHg in the Age 40 Program and a diastolic blood pressure of at least 90 mmHg in the ACE 1950 Study first visit.

**Supplemental Table S7: Associations Between Blood Pressure Trajectories Including Antihypertensive Treatment Between Age 40 and the Mid-60s and Neuroimaging Features of Cerebral Small Vessel Disease at Age 70 Years, Without Adjustment of Physical Activity**

|  |  |  | **Lacunes (logistic regression)** | | | | **Fazekas scale (ordinal regression)** | |  |  |
| --- | --- | --- | --- | --- | --- | --- | --- | --- | --- | --- |
|  | **Total (%)** | | | **n^a^ (%)** | **OR^b^ (95% CI)** | **aOR^c^ (95% CI)** | **OR^b^ (95% CI)** | **aOR^c^ (95% CI)** |  |  |
| **Systolic blood pressure trajectories** |  | | |  |  |  |  |  | Diastolic BP at age 40,  mmHg, mean±SD | Diastolic BP in mid-60s,  mmHg, mean±SD |
| Non-elevated throughout | 36 (15) | | | 2 (6) | 1 (ref) | 1 (ref) | 1 (ref) | 1 (ref) | 69.1±5.2 | 67.1±7.6 |
| High elevated to hypertension | 91 (37) | | | 12 (13) | 2.46 (0.52-11.71) | 1.36 (0.26-7.22) | 2.65 (1.21-5.82) | 2.75 (1.16-6.53) | 83.1±7.5 | 80.8±9.5 |
| Hypertension throughout | 116 (48) | | | 20 (17) | 3.39 (0.75-15.40) | 1.53 (0.29-8.03) | 2.38 (1.11-5.13) | 2.12 (0.90-4.99) | 87.9±8.3 | 83.3±9.6 |
| Total | 243 (100) | | | 34 (14) | - | - | - | - | 83.3±9.9 | 80.0±10.8 |
| **Diastolic blood pressure trajectories** |  | | |  |  |  |  |  | Systolic BP at age 40,  mmHg, mean±SD | Systolic BP in mid-60s,  mmHg, mean±SD |
| Non-elevated throughout | 21 (11) | | | 2 (10) | 1 (ref) | 1 (ref) | 1 (ref) | 1 (ref) | 111.6±11.2 | 119.3±11.2 |
| Elevated to hypertension | 113 (59) | | | 20 (18) | 2.10 (0.45-9.84) | 1.08 (0.19-6.24) | 2.23 (0.90-5.52) | 2.84 (0.99-8.18) | 136.8±10.6 | 144.2±18.7 |
| Hypertension throughout | 59 (31) | | | 11 (19) | 2.32 (0.46-11.63) | 1.38 (0.22-8.58) | 2.37 (0.90-6.28) | 2.71 (0.89-8.26) | 147.3±10.4 | 146.4±17.1 |
| Total | 193 (100) | | | 33 (17) | - | - | - | - | 137.3±14.6 | 142.2±19.3 |

^a^ Number of individuals with lacunes; ^b^ Adjusted for age at brain magnetic resonance imaging (MRI); ^c^ Adjusted for sex, education (ACE 1950), smoking (ACE 1950), hypercholesterolemia (ACE 1950), history of stroke (ACE 1950), diabetes (ACE 1950), and age at brain MRI. *Systolic blood pressure: Non-elevated throughout,* no more than 120 mmHg in the Age 40 Program and in the ACE 1950 Study first visit; *High elevated to hypertension*, blood pressure of 130-139 mmHg in the Age 40 Program and at least 140 mmHg or use of antihypertensive medication in the ACE 1950 Study first visit; *Hypertension throughout*, blood pressure of at least 140 mmHg or self-reported hypertension in the Age 40 Program and a systolic blood pressure of at least 140 mmHg or the use of antihypertensive medication in the ACE 1950 Study first visit. *Diastolic blood pressure: Non-elevated throughout*, blood pressure of no more than 70 mmHg in the Age 40 Program and in the ACE 1950 Study first visit; *Elevated to Hypertension*, blood pressure of 70-89 mmHg in the Age 40 Program and at least 90 mmHg or the use of antihypertensive medication in the ACE 1950 Study first visit; *Hypertension throughout*, blood pressure of at least 90 mmHg or self-reported hypertension in the Age 40 Program and a diastolic blood pressure of at least 90 mmHg or the use of antihypertensive medication in the ACE 1950 Study first visit.
